# Investigating the Associations of Constructivist Beliefs and Classroom Climate on Teachers' Self-Efficacy Among Australian Secondary Mathematics Teachers

**DOI:** 10.3389/fpsyg.2021.626271

**Published:** 2021-03-03

**Authors:** Fang Guangbao, Teo Timothy

**Affiliations:** ^1^School of Teacher Education, Jiangxi Normal University Science and Technology College, Gongqingcheng, China; ^2^Faculty of Education, Monash University, Melbourne, VIC, Australia; ^3^School of Education, Murdoch University, Perth, WA, Australia

**Keywords:** constructivist beliefs, teachers self-efficacy, Australia, mathematics teachers, classroom climate

## Abstract

This study examines the associations of constructivist beliefs and classroom climate on teachers' self-efficacy in instruction, classroom management, and student engagement among Australian secondary mathematics teachers. To do this, it uses the integrated model of teachers' self-efficacy with the concept of analysis of teaching tasks. The study uses structural equation modeling to analyze data from 495 mathematics teachers in the Teaching and Learning International Survey (TALIS) 2013. The results reveal the integrated model is a valid theoretical framework to explain Australian secondary mathematics teachers' self-efficacy. Teachers' constructivist beliefs and classroom climate are positively and statistically significantly related to teachers' self-efficacy in instruction, classroom management and student engagement. In contrast, constructivist beliefs have no significant correlation with classroom climate.

## Introduction

Teachers' self-efficacy has been shown to be related to valued educational processes and outcomes (Ashton and Webb, 1986b; Ross, 1992; Tschannen-Moran and Woolfolk, 2001; Betoret, 2009; Tran et al., 2012). Given this consideration, how to improve teachers' self-efficacy is viewed with increasing importance by school leaders, teachers, policymakers, and educational researchers.

**Figure 1:**
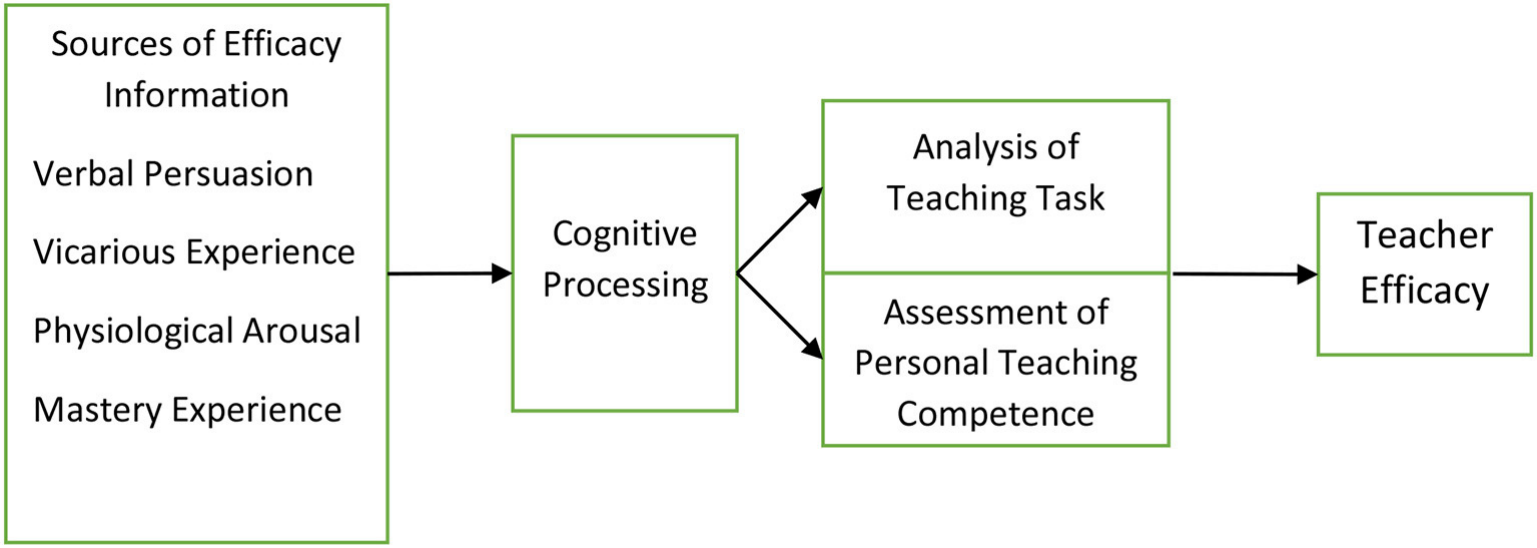
The integrated model of teacher efficacy. Sources from Tschannen-Moran et al. (1998).

According to the social cognitive theory, self-efficacy has four vital sources: mastery experiences, verbal persuasion, vicarious experiences, and physiological and affective states (Bandura, 1997). However, Bandura's sources of self-efficacy theory have been widely discussed to explain teachers' self-efficacy development (Charalambous et al., 2008; Chang, 2009), while it was proposed based on the de-scenario perspective. As a result, a new integrated model of teachers' self-efficacy was proposed (Tschannen-Moran et al., 1998). By adding three mediators, namely, TCP, teachers' cognitive processing; ATT, analysis of the teaching task; APTC, the assessment of personal teaching competence, the integrated model of teachers' self-efficacy could explain more effectively how the four sources influence self-efficacy (see Figure 1).

According to Tschannen-Moran et al. (1998, p. 231), the ATT stipulates that “teachers must assess what will be required of them in the anticipated teaching situation,” including the factors which relate to students, instruction, or classroom management. Although the obscured definition leads to limited substantial progress on the research of sources on teachers' self-efficacy (Philippou and Pantziara, 2015), the integrated model provides insightful implications on discussing the sources of teachers' self-efficacy based on the perspective of teachers' beliefs toward instruction, values on students and perceptions on classroom management.

A new concept in mathematics education, called “teachers” competence of analyzing situation contexts' (TCASC) (Kuntze et al., 2015) shares a similar meaning with the ATT, which refers to an “awareness-driven, knowledge-based process which connects the subject of analysis with relevant criterion knowledge and is marked by criteria-based explanation and argumentation” (Kuntze et al., 2015, p. 3,214). In their recent study, Kuntze and Friesen (2018) further explained that factors related to teaching knowledge and materials, classroom environment, and students are all included within this concept (TCASC).

The concept of ATT and TCASC share a similarity in analyzing teaching context-based on three aspects: teaching, classroom management, and students. This similarity works as a bridge to justify utilizing the integrated model of teachers' self-efficacy among mathematics teachers. There is limited evidence-based research exploring the self-efficacy of mathematics teachers. Furthermore, in this study, the similarity was operationalized with two specific concepts: teachers' constructivist beliefs and classroom climate.

To fill the theoretical gap in mathematics education identified above, the data of Australian mathematics teachers from the Teaching and Learning International Survey (TALIS) 2013 are utilized to address the following three research questions: (1) To what extent are Australian mathematics teachers' constructivist beliefs associated with their perceptions of classroom climate? (2) To what extent are Australian mathematics teachers' constructivist beliefs associated with their self-efficacies? (3) To what extent are Australian mathematics teachers' perceptions of classroom climate associated with their self-efficacies?

## Literature Review

### Teachers' Constructivist Beliefs and Classroom Climate

Teachers' constructivist beliefs refer to teachers' perceptions on their teaching; that is, the beliefs held by teachers about their preferred ways of teaching and learning (Aypay, 2011). In the TALIS 2013 theoretical framework, constructivist beliefs form that part of teachers' background (e.g., professional training/experience) (OECD, 2013a, p. 151) which is mainly concerned with “both about the ways [teachers] believe students learn best and how they as teachers might facilitate this learning” (OECD, 2013a, p. 165). Many studies (Cheng et al., 2009; Baeten et al., 2013) also explored the contents of constructivist beliefs, such as using student-centered teaching methods, teachers acting as facilitators in student learning, and students' need to engage in learning positively. Notably, because of the different philosophical foundations of constructivist theory, constructivist beliefs can have different preferences. The cognitive constructivist theory focuses on students' construction of knowledge and the constructing process itself, while the social constructivist theory focuses on students obtaining knowledge by participating in meaningful social practices (Wildman, 2008).

Therefore, when teachers hold constructivist beliefs toward teaching, they are more likely to adopt student-centered teaching methods rather than teacher-structured teaching methods. Specifically, students do not take absorbing knowledge for granted but actively participate in learning and create knowledge. Meanwhile, during the teaching process, teachers' act as facilitators, rather than controllers, and their main concern is to help students learn effectively rather than maintain their authority in the classroom.

The classroom climate is defined as the instructional and social-emotional environments students live in Babad (2009). In the TALIS 2013 theoretical framework, the classroom climate concept has desirable and undesirable measures, including maintaining order, controlling and managing student misbehaviors, and building a positive learning atmosphere (Fackler and Malmberg, 2016; Fackler et al., 2021). This operational definition in TALIS 2013 shares a similarity with the dynamic cascade model of classroom discipline climate, including the positive and preventive measures at the same time (Stefanich and Bell, 1985).

Within the teaching context, teachers who hold constructivist beliefs focus on helping students to participate in learning actively rather than on their authority and control of students in the classroom. Moreover, a good classroom climate means teachers exercise low control over students' behaviors and provide supportive conditions for students' learning. Thus, this study assumes that teachers' constructivist beliefs would be associated with the classroom climate. Similarly, Rubie-Davies and Peterson (2011) argued that teachers' beliefs positively influence the classroom climate. Based on the above discussion, the following hypothesis was formulated:

H1: The investigated Australian secondary mathematics teachers' constructivist beliefs are positively associated with their classroom climate perceptions.

### Constructivist Beliefs and Teachers' Self-Efficacy

Teachers' self-efficacy has been widely regarded as an influential factor that predict students' outcomes (Peters, 2013; Cheema and Kitsantas, 2014) and instructional practice (Holzberger et al., 2014; Klassen and Tze, 2014). However, the discussions on teachers' self-efficacy imply reciprocal relations with classroom factors (Tschannen-Moran et al., 1998; Ross et al., 1999; Choi et al., 2019). That is to say, teachers' self-efficacy would be an outcome, which may influence by classroom-related factors. Thus, in this study, teachers' self-efficacy has been operationalised as the outcome variable with an aim to explore its relationships with teachers' constructivist beliefs and classroom climate.

The statistically significant relationship between teachers' self-efficacy and teachers' constructivist beliefs has been reported in several studies. These included studies that analyzed TALIS (2008 and 2013) which revealed that teachers who hold a higher level of constructivist beliefs were more likely to have a higher self-efficacy level (Vieluf et al., 2012; OECD, 2013a). This positive relationship still existed, even after controlling for other factors that exert influences on teachers' constructivist beliefs using multilevel designs (Fackler and Malmberg, 2016; Fackler et al., 2021). Moreover, by using the difference-in-difference technique and instrumental variable, casual research indicated that teachers' constructivist beliefs and practices could strongly influence teachers' self-efficacy as well (Choi et al., 2019).

The relationship between teachers' constructivist beliefs and their self-efficacy were also confirmed among the other teachers' groups. For instance, among early childhood teachers, Cobanoglu and Capa-Aydin (2015) found that those who held higher constructivist beliefs had higher efficacy levels when engaging with students and using various instructional strategies. Similarly, among preservice teachers, Dunn and Rakes (2011) revealed that teachers' self-efficacy was significantly related to their learner-centered beliefs. This finding was echoed in the research of Temiz and Topcu (2013). In addition, Gürbüztürk and Sad (2009) found positive correlations between student teachers' constructivist beliefs and their self-efficacy in student engagement, while traditional teaching beliefs positive correlate with efficacy in classroom management, instruction, and the overall efficacy. Nie et al. (2013) reported that the association between teachers' self-efficacy and constructivist instruction was stronger than the relationship between teachers' self-efficacy and didactic instruction.

The above discussion illustrated the relationship between teachers' constructivist beliefs and their self-efficacy, and highlighted the paucity of the research exploring this relationship among secondary mathematics teachers. In this study, we argue that mathematics teachers' constructivist beliefs are positively associated with their self-efficacy. Based on the above discussion, the following three hypotheses are proposed:

H2: The investigated Australian secondary mathematics teachers' constructivist beliefs are positively associated with their self-efficacy in classroom management.
H3: The investigated Australian secondary mathematics teachers' constructivist beliefs are positively associated with their self-efficacy in instruction.
H4: The investigated Australian secondary mathematics teachers' constructivist beliefs are positively associated with their self-efficacy in student engagement.

### Classroom Climate and Teachers' Self-Efficacy

As mentioned earlier, classroom climate was defined as maintaining teaching order, controlling students' misbehaviors, and providing support to students in the TALIS 2013 framework. Thus, classroom climate would be perceived as the critical strategy that teachers adapt to address classroom management. This study extends the review of the relationship between classroom climate and teachers' self-efficacy by adding the relations between classroom management and teachers' self-efficacy.

Some studies explored the relationship of classroom climate and teachers' self-efficacy directly (Coladarci, 1992; Fackler and Malmberg, 2016; Perera et al., 2019; Fackler et al., 2021). A controversial conclusion was reached by Fackler and Malmberg (2016) who found no statistically significant relationship between classroom climate and teachers' self-efficacy by analyzing the TALIS 2008 data. However, using multilevel modeling (Fackler et al., 2021) on the TALIS 2013 data, this study obtained a contrary finding in support of a relationship between classroom climate and teachers' self-efficacy, in alignment with Perera et al. (2019) who found that classroom climate was only one predictor of teachers' self-efficacy.

Some studies investigated the relationship between classroom management and teachers' self-efficacy (Ashton and Webb, 1986a,b; Woolfolk and Hoy, 1990; Woolfolk et al., 1990; Gencer and Cakiroglu, 2007). For example, Ashton and Webb (1986a) revealed that teachers' self-efficacy was significantly related to teachers' classroom management. Similarly, Rimm-Kauffman and Sawyer (2004) claimed that teachers who held higher self-efficacy would have higher classroom management requirements. Specifically, Woolfolk et al. (1990) reported a negative correlation between teachers' teaching efficacy and their attitudes toward student control. These studies inferred that the more teachers have a strong custodial orientation toward pupils, the less they would favor increased student autonomy.

Similarly, Woolfolk and Hoy (1990) identified a negative correlation among prospective teachers' teaching efficacy, personal efficacy, and bureaucratic orientation, while only teaching efficacy has a significant correlation with student control ideology. In contrast, by investigating 584 preservice science teachers, Gencer and Cakiroglu (2007) found a positive correlation between teaching self-efficacy and classroom management. In other words, preservice science teachers who had strong confidence in teaching science showed higher proclivity in controlling their students.

Accordingly, we argue that classroom climate may have direct relations with teachers' self-efficacy. So, the following three hypotheses are proposed:

H5: Classroom climate positively correlates with the participated Australian secondary mathematics teachers' self-efficacy in classroom management.
H6: Classroom climate positively correlates with the participated Australian secondary mathematics teachers' self-efficacy in instruction.
H7: Classroom climate positively correlates with the participated Australian secondary mathematics teachers' self-efficacy in student engagement.

**Figure 2:**
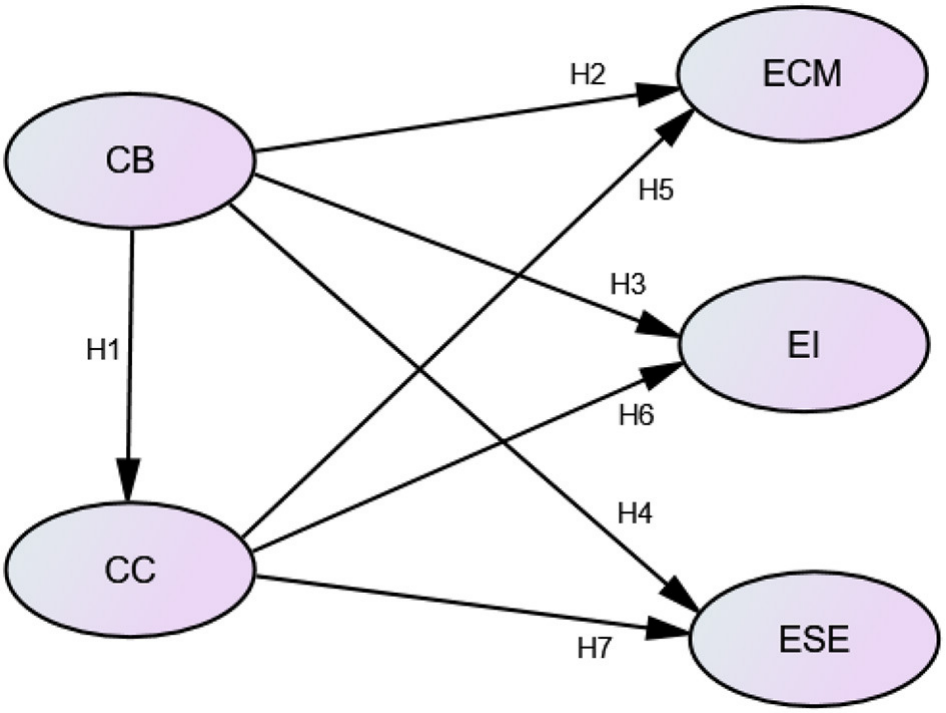
Research model. CB, constructivist beliefs; CC, classroom climate; ECM, efficacy in classroom management; EI, efficacy in instruction; ESE, efficacy in student engagement.

The relationships among the five constructs are illustrated in Figure 2.

## Methodology

### Data Source

The current study uses the Australian mathematics teachers' sample data from the TALIS 2013, on ISCED 2 level, for two reasons: (1) this survey focuses on teachers' pedagogical beliefs and self-efficacy in the classroom; thus, it could offer sufficient information for this research; (2) the data was collected with scientific rigor, adopting a stratified, two-stage probability sampling design. A total of 495 mathematics teachers, including 229 females (46.3%) and 266 males (53.7%) from 120 secondary schools in Australia, participated in the survey. On average, each school cluster has 3.925 mathematics teachers. Participants' teaching experience ranged from 0 to 43 years, with an average duration of 16.86 years (SD = 11.12). In the TALIS 2013 Australian data, there were 1212 females (58.9%) and 847 males (41.1%), and teachers' teaching experience ranged from 0 to 48, with an average teaching year of 16.59 (SD = 11.11). Compared with the TALIS 2013 Australian data, the gender composition in this study may have a little difference, while the teaching experience is extremely similar.

### Key Measures

This study's five constructs are assessed by three questions in the teachers' questionnaire in TALIS 2013.

Teachers' constructivist belief (CB) was measured with four items. These four items concern the topics of teachers' role in teaching, students' role in learning, and the methods of learning. The responses were answered on a four-point Likert scale, from 1 = strongly disagree to 4 = strongly agree. According to the technical report of TALIS 2013, the coefficient Alpha of Australian teachers' constructivist beliefs was 0.705, and the CFA results indicated a good model fit of data (CFI = 0.998, TLI = 0.994, RMSEA = 0.018, and SRMR = 0.009) (OECD, 2013b, p. 240–242).

Teachers' classroom climate (CC) was assessed with four items, while three items (TT2G41A, TT2G41C, and TT2G41D) being reverse-coded. The topics reflected by items for CC include controlling students, creating a supportive learning atmosphere, maintaining order, and preventing misbehavior. The responses also were answered on a four-point Likert scale, from 1 = strongly disagree to 4 = strongly agree. The coefficient Alpha of Australian teachers' classroom climate was 0.878, and the CFA results indicated good model fits of data (CFI = 0.999, TLI = 0.998, RMSEA = 0.012, and SRMR = 0.007) (OECD, 2013b, p. 232–234).

Teachers' perceptions of self-efficacy in the classroom was further divided into three sub-parts: efficacy in classroom management (ECM), efficacy in instruction (EI), and efficacy in student engagement (ESE). The construct of teachers' self-efficacy was measured with 12 items. Again, all these items were answered on a four-point Likert scale: 1 = not at all, 2 = to some extent, 3 = quite a bit, and 4 = a lot. The Coefficient Alpha of Australian teachers' ECM, EI, and ESE was 0.847, 0.787, and 0.842, respectively. The model fits Australian teachers' self-efficacy, which included all three sub-constructs, were CFI = 0.942, TLI = 0.925, RMSEA = 0.060, and SRMR = 0.048 (OECD, 2013b, p. 197–200).

### Data Analysis

Considering all participating mathematics teachers are clustered in schools and their perceptions of self-efficacy would have a high correlation, this study ran a two-level null model to compute the intra-class correlation (ICC) to decide whether or not a multilevel model was needed. The ICC values range from 0.022 to 0.059, which were less than the suggested rule of thumb (0.059) (Peugh, 2010; Huang, 2018) with one exception equal to 0.059. Furthermore, we computed the design effect values, which ranged from 1.064 to 1.172, less than the recommended value of 2 (Lai and Kwok, 2015). Finally, since each cluster in our data has only 3.952 mathematics teachers, which is far <30 (Huang, 2018), this study had adopted a single-level structural equation modeling to analyze the study data.

Following Anderson and Gerbing (1988), the two-step analytical strategy was adopted using SPSS 25.0 and AMOS 25.0 software. First, the reliability and validity of the measurement model was estimated. As noted above, the OECD technical report offers the Coefficient Alpha and the model fit of the constructivist beliefs, classroom climate and teachers' self-efficacy. In addition, this study computed the item factor loadings, Cronbach's Alpha, squared multiple correlations (SMC), composite reliability (CR), and average variance extracted (AVE), based on two considerations: (1) only mathematics teachers were selected, and (2) the construct of self-efficacy had been divided into three parts in this study. Second, the structural relationships among these three constructs were assessed using maximum likelihood estimation.

## Results

### Descriptive Analysis

The means and standard deviations of all constructs are shown in Table 1. The mean values of item measuring constructivist beliefs (CB) range from 2.81 to 3.26 (SD ranges from 0.587 to 0.715), indicating that the Australian teachers had reported a relatively positive response to these beliefs. For the item measuring classroom disciplinary climate (CDC), the mean values range from 2.70 to 2.96 (SD ranges from 0.784 to 0.86). These results reveal lower perceptions of classroom disciplinary climate. For the self-efficacy variables, the results indicated a slightly lower mean value of efficacy in student engagement, ranging from 2.80 to 3.27 (SD ranges from 0.688 to 0.752), and the mean values of the efficacy in instruction range from 3.12 to 3.54 (SD ranges from 0.611 to 0.749). Similarly, the mean values of the efficacy in classroom management range from 3.14 to 3.49 (SD ranges from 0.649 to 0.740).

**Table 1:** Descriptive results of the constructs.

**Constructs**	**Items**	**Means**	**Std. deviations**
Constructivist beliefs (CB)	CB1	3.26	0.587
	CB2	2.81	0.705
	CB3	3.18	0.623
	CB4	3.02	0.715
Classroom climate (CC)	CC1	2.95	0.798
	CC2	2.70	0.784
	CC3	2.88	0.860
	CC4	2.96	0.836
Efficacy in student engagement (ESE)	ESE1	3.27	0.688
	ESE2	3.16	0.752
	ESE3	2.80	0.750
	ESE4	2.99	0.723
Efficacy in instruction (EI)	EI1	3.20	0.672
	EI2	3.16	0.723
	EI3	3.54	0.611
	EI4	3.12	0.749
Efficacy in classroom management (ECM)	ECM1	3.21	0.740
	ECM2	3.49	0.649
	ECM3	3.31	0.682
	ECM4	3.14	0.737

Indices of normality of the data for these five constructs was examined. Based on the suggestion of Hair et al. (2010), a skewness-kurtosis test was conducted to assess whether the data follows the rule of normal distribution. The values of skewness and kurtosis range from −1.025 to 0.132, and from −1.083 to 0.638, respectively, which fall within the recommended range of |3| and |8| (Kline, 2010).

### Test for Measurement Model

Before conducting the confirmatory factor analysis (CFA), the multivariate normality was assessed using a Mardia's coefficient. In this study, the obtained value is 52.518, which was less than the value 440 recommended by Raykov and Marcoulides (2008), computed by the formula [*p* (*p*+2)] where *p* refers to the total number of items. Therefore, this raw data met the multivariate normality requirement and could be used to conduct the CFA analysis. The Cronbach's Alpha of CB is 0.687, which is very close to 0.70, while the remaining four constructs' Cronbach's Alpha is over 0.70. Because Cronbach's Alpha is sensitive to the sample size, composite reliability (CR) and average variance extracted (AVE) were also assessed to establish item reliability. According to the results in Table 2, all constructs' CR and AVE met the requirements of 0.70 (Nunnally and Bernstein, 1994; Gefen et al., 2000) and 0.50 (Fornell and Laker, 1981), respectively. Thus, all the items of these five constructs have acceptable reliability. The factor loadings of all items range from 0.665 to 0.906 (see Table 2), larger than the recommended value of 0.50 (Hair et al., 2010). This indicates that all the items of the five constructs have good content validity. The SMC of CB, CDC, ESE, ECM, and EI range from 0.442 to 0.604, from 0.656 to 0.821, from 0.546 to 0.787, from 0.613 to 0.757, from 0.444 to 0.709, respectively. This means these items explain at least 40% of the variance in each construct. To assess the discriminant validity between constructs, the AVE value must be superior to the squared correlation between the constructs (Fornell and Laker, 1981). As reported in Table 3, the AVE (bold numbers in the diagonal) for all constructs are larger than the squared correlations between the constructs, which suggests that the constructs' measurements had adequate discriminate validity.

**Table 2:** Summary of measurement model.

**Constructs**	**Items**	**Cronbach's Alpha**	**Factor loading**	**SMC**	**CR**	**AVE**
Constructivist beliefs (CB)	CB1	0.687	0.665	0.442	0.811	0.519
	CB2		0.777	0.604		
	CB3		0.750	0.563		
	CB4		0.683	0.466		
Classroom climate (CC)	CC1	0.886	0.852	0.726	0.921	0.745
	CC2		0.810	0.656		
	CC3		0.906	0.821		
	CC4		0.881	0.776		
Efficacy in student engagement (ESE)	ESE1	0.843	0.853	0.728	0.896	0.683
	ESE2		0.887	0.787		
	ESE3		0.819	0.671		
	ESE4		0.739	0.546		
Efficacy in classroom management (ECM)	ECM1	0.848	0.870	0.757	0.898	0.689
	ECM2		0.783	0.613		
	ECM3		0.850	0.723		
	ECM4		0.814	0.663		
Efficacy in instruction (EI)	EI1	0.766	0.666	0.444	0.852	0.592
	EI2		0.766	0.587		
	EI3		0.792	0.627		
	EI4		0.842	0.709		

*SMC, Square multiple correlation; CR, Composite reliability; AVE, Average variance extracted*.

**Table 3:** AVE and squared correlation between the constructs.

	**CB**	**CC**	**EI**	**ESE**	**ECM**
CB	**0.519**				
CC	0.0002	**0.745**			
EI	0.028	0.094	**0.592**		
ESE	0.058	0.138	0.503	**0.683**	
ECM	0.002	0.133	0.575	0.415	**0.689**

*CB, Constructivist beliefs; CC, Classroom climate; ECM, Efficacy in classroom management; EI, Efficacy in instruction; ESE, Efficacy in student engagement*.

### Test for Structural Model

As mentioned, a series of indices were used to assess the structural model fit. The Chi-square of the minimum fit function was 454.829, a *p*-value below 0.001, while the ratio of the Chi-square of the minimum fit function to its degree of freedom was 2.879, which was <3.0 as suggested by Carmines and Mclver (1981). The value of the Comparative Fit Index (CFI) and Tucker-Lewis Index (TLI) was 0.926 and 0.902, respectively, which was larger than 0.90, as suggested by Hair et al. (2010). The value of root means the square error of approximation (RMSEA) was 0.063, <0.080. Thus, the structural model was assessed to have a good model fit.

Table 4 reports the association of constructivist beliefs and classroom climate. The constructivist beliefs were shown to have no statistically significant relationship with classroom climate while both constructivist beliefs and classroom climate shared statistically significant positive correlations with teachers' three efficacies in the classroom (see Table 4).

**Table 4:** Summary of the structural model.

**Hypotheses**	**Relationships**	**Path coefficient**	***t* value**	**Results**
H1	CB——>CC	−0.04	−0.222	Not supported
H2	CB——>ECM	0.72^***^	4.59	Supported
H3	CB——>EI	0.88^***^	4.483	Supported
H4	CB——>ESE	0.69^***^	4.463	Supported
H5	CC——>ECM	0.40^**^	2.833	Supported
H6	CC——>EI	0.35^**^	2.008	Supported
H7	CC——>ESE	0.40^**^	2.899	Supported

*CB, Constructivist beliefs; CC, Classroom climate; ECM, Efficacy in classroom management; EI, Efficacy in instruction; ESE, Efficacy in student engagement*.

*** p < 0.001;

** *p < 0.05*.

**Figure 3:**
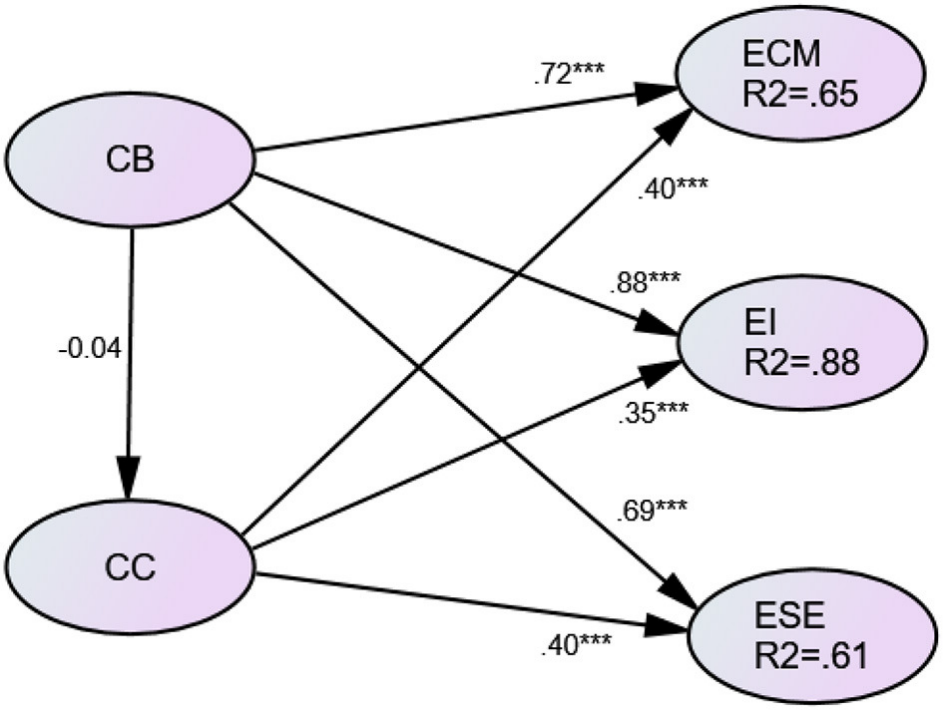
Research model results. CB, constructivist beliefs; CC, classroom climate; ECM, efficacy in classroom management; EI, efficacy in instruction; ESE, efficacy in student engagement. ***p < 0.001.

Efficacy in classroom management was significantly related to teachers' constructivist beliefs and classroom climate, resulting in a determination coefficient (R^2^) of 0.65. Efficacy in instruction and efficacy in student engagement had 88% and 61% of its variance explained, respectively, and was correlated with constructivist beliefs and classroom climate (see Figure 3).

## Discussion and Conclusion

This study investigates the associations of teachers' constructivist beliefs and perceptions of classroom climate with their self-efficacies in the classroom, using data from Australian secondary mathematics teachers. The results support the integrated model of teachers' self-efficacy (Tschannen-Moran et al., 1998). The sampled Australian mathematics teachers' classroom efficacies was positively related to their constructivist beliefs and classroom climate.

Constructivist beliefs (CB) have no statistically significant association with classroom climate (CC) in the given sample. This finding is not consistent with the result of Rubie-Davies and Peterson (2011). We explain this inconsistent result on fronts. First, the factor loadings and Cronbach's Alpha value of constructivist beliefs are less than the other constructs, which suggest the presence of measurement errors in measuring constructivist beliefs. Second, teachers' constructivist beliefs are not equal to the practical constructivist behaviors, which means that, teachers who hold constructivist beliefs may not apply them in their teaching. This finding may indicate a lack of direct relationship between teachers' constructivist beliefs and their behaviors directly related to classroom climate.

Among the correlation of constructivist beliefs on classroom efficacies, constructivist beliefs have the largest association (0.880) with instruction efficacy. This is consistent with former research findings (Nie et al., 2013; Choi et al., 2019). Constructivist beliefs were statistically significantly related to the efficacy in classroom management and efficacy in student engagement with effect sizes of 0.72 and 0.69, respectively. Consistent with an earlier study (Christophersen et al., 2016), the significance of constructivist beliefs indicates that, if teachers prefer to use student-centered teaching method or have pedagogical knowledge of how to appeal to students' interests and attention, they tend to have high expectations and believe they can do a good job with classroom management, student engagement and instruction.

Classroom climate is found to have a significantly positive relationship with efficacy in classroom management, instruction, and student engagement, with effect sizes of 0.40, 0.35, and 0.40, respectively. This contrasts with former research (Christophersen et al., 2016), which indicates that when teachers create or prefer a positive climate, they are more likely to have a higher efficacy level within the classroom. This inconsistent result may explain the methods of measuring classroom climate. When the study measured the classroom climate negatively, teachers' efficacy may negatively correlate with their classroom climate perceptions or behaviors.

This study contributes to the theory of the integrated model of teachers' self-efficacy (Tschannen-Moran et al., 1998) by operationalizing the analysis of teaching task (ATT) with two constructs: constructivist beliefs and classroom climate. In addition, we extended the scope of research to the mathematics teachers and explored how their constructivist beliefs and classroom climate were related to teachers' self-efficacy in instruction, classroom management and student engagement. This study uses constructivist beliefs and classroom climate with good psychometric properties to explain mathematics teachers' self-efficacy in Australia. The results show supportive evidence for constructivist beliefs and classroom climate in explaining mathematics teachers' self-efficacies, with huge explanatory power. We find that teachers' constructivist beliefs have no significant correlation with classroom climate, while both constructivist beliefs and classroom disciplinary climate have statistically significant positive associations with Australian secondary mathematics teachers' self-efficacy in instruction, classroom management, and student engagement.

## Implications and Future Research

### Implications for Theory and Practice

This study contributes to the understanding of the sources of teachers' self-efficacy by exploring the content of analyses of teaching tasks based on an integrated model in the context of Australian secondary mathematics teachers. Importantly, this study uses empirical evidence to support the findings of a positive association of teachers' constructivist beliefs and classroom climate with teachers' self-efficacy in instruction, classroom management, and student engagement.

In addition to the theoretical contribution, this study also informs school leaders and teacher educators of the importance of teachers' constructivist beliefs and classroom climate. First of all, school leaders and teacher educators should help teachers incorporate constructivist learning theory and formulate a positive classroom climate to increase their confidence in classroom management, instruction, and student engagement, in the teachers' professional development programs and initial teacher education programs. Simultaneously, school reforms generally require teachers to refresh their instructional beliefs and judgment skills about their teaching (Charalambous and Philippou, 2010). Consistent with these requirements of school reforms, teachers' constructivist beliefs, which are more focused on student-centered strategies, have been widely adopting by teachers and educational policymakers. However, this study reveals that the sampled Australian mathematics teachers' perceptions or beliefs would not ensure their behaviors. Thus, for teacher educators, we suggest that, apart from educating mathematics teachers in latest beliefs or theories, school leaders should advocate for the change in actual teaching behaviors.

### Limitations and Future Research

Some limitations are found in this study. First, the analyzed data are taken from TALIS 2013 Australian teachers' data and these are self-reports collected using a cross-sectional methodology. Hence, causality cannot be implied. Moreover, the concept of analysis of the teaching task is quite broad and may cover other concepts in addition to constructivist beliefs and classroom climate. Also, this study was restricted to mathematics teachers. As teachers' self-efficacy may differ across subjects, this study's findings cannot be generalized to other subject teachers. For future studies, researchers should adopt a longitudinal or experimental design to examine the causal effects to examine to under the relationships among the constructs included in this study.

## Data Availability Statement

Publicly available datasets were analyzed in this study. This data can be found here: OECD+http://www.oecd.org/education/school/talis-2013-results.htm.

## Ethics Statement

Ethical review and approval was not required for the study on human participants in accordance with the local legislation and institutional requirements. Written informed consent for participation was not required for this study in accordance with the national legislation and the institutional requirements.

## Author Contributions

FG was in charge of this paper and write the whole section and pay for the publication fees. TT was in charge of providing professional revising suggestions. Both authors contributed to the article and approved the submitted version.

## Conflict of Interest

The authors declare that the research was conducted in the absence of any commercial or financial relationships that could be construed as a potential conflict of interest.
